# Rheb-TOR signaling promotes protein synthesis, but not glucose or amino acid import, in *Drosophila*

**DOI:** 10.1186/1741-7007-5-10

**Published:** 2007-03-19

**Authors:** Dayna J Hall, Savraj S Grewal, Aida Flor A de la Cruz, Bruce A Edgar

**Affiliations:** 1 Division of Basic Sciences, Fred Hutchinson Cancer Research Center, 1100 Fairview Avenue North, Seattle, Washington 98109, USA

## Abstract

**Background:**

The Ras-related GTPase, Rheb, regulates the growth of animal cells. Genetic and biochemical tests place Rheb upstream of the target of rapamycin (TOR) protein kinase, and downstream of the tuberous sclerosis complex (TSC1/TSC2) and the insulin-signaling pathway. TOR activity is regulated by nutritional cues, suggesting that Rheb might either control, or respond to, nutrient availability.

**Results:**

We show that Rheb and TOR do not promote the import of glucose, bulk amino acids, or arginine in *Drosophila *S2 cells, but that both gene products are important regulators of ribosome biogenesis, protein synthesis, and cell size. S2 cell size, protein synthesis, and glucose import were largely insensitive to manipulations of insulin signaling components, suggesting that cellular energy levels and TOR activity can be maintained through insulin/PI3K-independent mechanisms in S2 cell culture. *In vivo *in *Drosophila *larvae, however, we found that insulin signaling can regulate protein synthesis, and thus may affect TOR activity.

**Conclusion:**

Rheb-TOR signaling controls S2 cell growth by promoting ribosome production and protein synthesis, but apparently not by direct effects on the import of amino acids or glucose. The effect of insulin signaling upon TOR activity varies according to cellular type and context.

## Background

Rheb (*R*as *h*omolog *e*nriched in *b*rain) is a small, highly conserved guanine triphosphatase (GTPase) that regulates cell growth in *Drosophila *and mammals. It has been demonstrated through genetic and biochemical analyses to function downstream of the tuberous sclerosis complex (TSC1/TSC2) and upstream of the conserved protein kinase, target of rapamycin (TOR; [1-3]), which regulates many processes important for cell growth including protein synthesis, ribosome biogenesis, autophagy, and (in yeast) amino acid import [4-10]. TSC2, a known tumor suppressor, acts as a GTPase-activating protein (GAP) for Rheb [11-18], and thus inhibits Rheb activity via direct molecular interaction. It remains less clear how Rheb activates TOR, or how TOR activity drives cell growth. In a previous publication [2], we discussed two possible mechanisms via which Rheb might mediate its downstream effects. The first possibility is that Rheb activates TOR indirectly by promoting nutrient import at the plasma membrane, and thereby increases intracellular nutrient and/or energy pools. As TOR activity can be regulated by both amino acid levels and cellular energy levels as provided by glucose [14,19,20], this might explain the dependence of TOR activity upon Rheb. This possibility is consistent with the ability of Rheb to promote cell growth in amino acid-starved *Drosophila *larvae; the ability of other related small GTPases, such as the Rab GTPases, to promote endocytosis and glucose import [21,22]; the presumed localization of Rheb at the plasma membrane [23]; and the ability of insulin signaling, which has been reported to modulate Rheb-GTP loading [12], to promote glucose import in many cell types [24]. It is also consistent with studies from yeast showing that TOR regulates amino acid permeases [25]. Alternatively, Rheb might activate TOR through direct molecular interactions [26-29]. This simpler possibility is more consistent with the inability of cells with mutations in the *TOR *gene to grow even when Rheb is overexpressed, and with the ability of overexpressed Rheb to activate TOR targets in amino acid-deprived cells [2]. In this report, we evaluate these two possible mechanisms, and present data consistent with the second possibility. We also delve into the relationship between insulin signaling and TOR, and conclude that the ability of insulin signaling to regulate TOR is dependent on cell type, and may vary within a given cell type as nutritional conditions change.

## Results

### S2 cells deficient in Rheb or TOR are reduced in size

**Figure 1:**
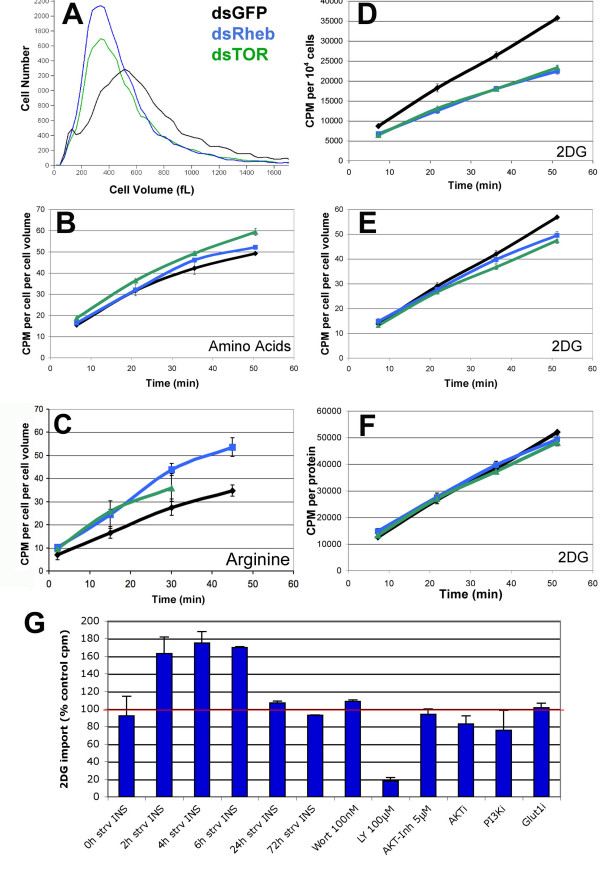
**Nutrient uptake in *Drosophila *S2 cells**. Representative data obtained from nutrient import experiments performed in triplicate following 72-hour treatments using dsRNA (RNAi) directed against Rheb, TOR, or GFP (control) to deplete the target transcript. (**A) **Histogram generated (Coulter Counter Z2 software) demonstrating that dsRheb-treated and dsTOR-treated cells are smaller than dsGFP-treated control cells.**(B) **Bulk amino-acid import normalized to cell number and volume shows that dsRheb-treated and dsTOR-treated cells are not deficient for general amino-acid import. (**C) **Arginine import normalized to cell number and volume shows that dsRheb-treated and dsTOR-treated cells have increased levels of arginine import. (**D) **Cellular 2-deoxyglucose import, when normalized to cell number, is significantly reduced in cells treated with dsRheb or dsTOR. When 2DG import is normalized using mean cellular volume **(E)**, or total cellular protein content **(F)**, 2DG import in dsRheb-treated or dsTOR-treated cells is comparable with controls. **G) **Effects of insulin signaling on 2DG import. Data represent percentage of control 2DG import at 25 (insulin experiments) or 35 minutes after 2DG addition. For the first six bars, growing subconfluent cells were deprived of serum for the indicated periods, and then stimulated with 10 μg/mL bovine insulin for 5 minutes prior to addition of 2DG. Wortmannin (Wort), LY294002 (LY), and 1L-6-Hydroxymethyl-chiro-inositol 2-(R)-2-O-methyl-3-O-octadecylcarbonate (AKT-Inh) were added to cells at the indicated doses 30 minutes before addition of 2DG, in the presence of serum. dsRNA directed against Akt (AKTi), PI3K (PI3Ki), or Glut1 (Glut1i) was applied 72 h beforre 2DG addition, also in the presence of serum. Error bars represent standard deviations of duplicate assays.

To test the aforementioned hypotheses,*Drosophila *Schneider line 2 (S2) cells were treated with double-stranded RNA (dsRNA, RNAi) to deplete Rheb, TSC2, or other relevant proteins that function in the insulin and TOR signal-transduction pathways. To confirm dsRNA knockdown of the targeted mRNAs, quantitative real time reverse transcription (RT) PCR or Northern analyses were performed (Additional file 1). Using a Beckman Coulter Counter to measure cell volumes, we noted that S2 cells subjected to 72 hours of dsRheb- or dsTOR-treatment were smaller than control cells (Figure 1A). This is consistent with previously published results from S2 cells and other systems, and is also seen *in vivo *in *Drosophila rheb*, *TOR*, or *s6k *(a TOR target) gene mutants [1,2,30,31].

### General amino acid import is not impaired in cells deficient in Rheb or TOR

To test the hypothesis that Rheb directly controls the cellular import of amino acids, we developed a simple amino-acid import assay. At 72 hours after treatment with dsRNA, cells were transferred to an isosmotic salt solution containing a mix of 15 tritiated amino acids at 22°C. At time-points following this, the cells were washed and lysed, and the amount of ^3^H in the lysates was determined using a scintillation counter. Time courses of cellular uptake of amino acids revealed that treatment of S2 cells with dsRNA directed against Rheb or TOR consistently caused a slight increase in amino-acid import relative to control cells, which were treated with dsRNA against GFP, when the data were normalized per cell volume (Fig 1B) or per total cellular protein (not shown). Similar assays were performed on cells treated for 30 min with rapamycin, an inhibitor of TOR, or LY294002, an inhibitor of PI3K as well as TOR. These short treatments did not change cell size, and detected no significant deficits in amino-acid import. Longer exposures to rapamycin (5–50 hrs) also had no detectable effect on amino-acid uptake. As a control for the biological relevance of the assay, we performed several import assays on ice at 0°C. This blocked ^3^H-amino acid uptake nearly completely, and the effect was rapidly reversed by restoring the cells to 22°C (data not shown). Overall, these results indicate that the activity of Rheb and TOR is not an important regulator of bulk amino-acid import. Hence, we suggest that the promotion of general amino-acid import is not likely to be a critical function of either Rheb or TOR in regulating S2 cell growth.

### Arginine import is increased, not reduced, in cells deficient in Rheb or TOR

Two recent reports suggest that TOR might control cell growth by specifically regulating the import of basic amino acids via a cationic amino-acid permease encoded by the *slimfast *(*slif*) gene [32,33]. These studies show that *slif *is required for normal rates of organ growth in flies, that its localization to the plasma membrane of adipose cells in the fat body is controlled by TOR activity, and that it is a limiting regulator of arginine import in S2 cells. The role of TOR in regulating arginine import, however, was not tested directly. To investigate the possibility that TOR regulates cell growth by specifically controlling the import of basic amino acids, we used RNAi to deplete Rheb or TOR and then measured the uptake of ^3^H-arginine in S2 cells. Experiments performed in triplicate indicated that rates of ^3^H-Arg uptake were not reduced, but instead showed a small but reproducible increase, in cells depleted of Rheb or TOR (Figure 1C). These data are inconsistent with models in which the growth effects of TOR are mediated via Slif and the import of basic amino acids, but they concur with the observation that arginine import is increased in yeast mutant for Rheb [34]. The basis of this effect is not clear; Rheb/TOR signaling might negatively regulate arginine importers, or the effect may be an indirect, compensatory response to reductions in another TOR-regulated process, such as protein synthesis (see below).

### Glucose import is not impaired in cells deficient in Rheb or TOR

Genetic epistasis tests and biochemical evidence place *Drosophila *Rheb downstream of the insulin receptor (InR), phosphoinositide-3-kinase (PI3K) and the oncogenic protein kinase, Akt (also known as PKB), in the insulin-signaling cascade [1-3,12,18,30]. In mammals, insulin signaling functions to enhance glucose import in skeletal muscle and adipose tissue [24,35]. Moreover, the small GTPases Rab11 and Rab4, as well as a Rab-GAP, have been implicated in mediating translocation of the glucose importer GLUT4 to the plasma membrane upon insulin stimulation [21,22]. The influence of Rheb on glucose uptake was therefore tested. S2 cells were treated with dsRNA directed against Rheb for 72 hours, after which time rates of cellular import of tritiated 2-deoxyglucose (2DG) were measured. 2DG is a glucose analog that enters the cell and is phosphorylated to 2-DG-6-phosphate. It is not metabolized further and hence accumulates intracellularly [36]. dsRheb-treated S2 cells had a consistent, significant reduction in cellular 2DG import compared with dsGFP-treated control cells (Figure 1D). Inhibition of TOR with either dsRNA or 16-hour treatment with rapamycin yielded similar results. However, when these 2DG import data were corrected for differences in cell size or protein content, no effects of either loss of TOR or Rheb were apparent (Figures 1E, F). Moreover, a time-course of inhibition of TOR by rapamycin demonstrated that a deficiency in 2DG import was not observed until there was also an observable change in cell size (data not shown). Therefore, the decreased 2DG uptake can be attributed to the reduction in cell size, provided that we assume that the number of glucose importers per cell drops proportionally to the loss of surface area. These findings weigh against models in which Rheb activates TOR by stimulating glucose import. Moreover, they imply that the decreased size of cells lacking Rheb or TOR cannot be attributed to decreased glucose or amino-acid import.

### Insulin signaling has only minor effects on glucose import

Insulin signaling is an important regulator of glucose import in some mammalian cell types [24,35], and *in vivo *studies suggest that this function is conserved in *Drosophila *[37,38]. It has been proposed that insulin signaling might promote TOR activity by increasing glucose import and thereby supporting ATP pools [5,39]. To assess this possibility in *Drosophila*, we measured rates of glucose import in S2 cells stimulated with 10 μg/mL bovine insulin after various periods of serum starvation. As shown in Figure 1G, insulin markedly stimulated glucose import only in cells that had been starved of serum (and the insulin within it) for short periods of time (2–6 hours). Insulin stimulation of S2 cells that had not been serum-starved, or that had been serum-starved for 24 hours or more, had no significant effect on rates of glucose import. This indicates that S2 cells are capable of importing glucose independently of their insulin-signaling activity. We also assessed, in serum-stimulated cells, the effects of inhibiting insulin-signaling components with inhibitors or RNAi. Short-term inhibition of insulin signaling was achieved with the PI3K inhibitor wortmannin (see [40,41]) or an AKT inhibitor (1L-6-Hydroxymethyl-chiro-inositol 2-(R)-2-O-methyl-3-O-octadecylcarbonate; Calbiochem), and long-term inhibition of insulin signaling was achieved using dsRNA directed against PI3K or Akt. None of these treatments had major effects on rates of glucose import (Figure 1G). We also used RNAi to assess the potential role of Glut1, the *Drosophila *glucose transporter most homologous to mammalian Glut4, which mediates insulin-dependent glucose import in fat and muscle [24,35]. Although the Glut1 gene is essential for viability *in vivo *in *Drosophila*, RNAi-mediated depletion of Glut1 in S2 cells had no detectable effect on glucose import (Figure 1G). All these results indicate that insulin signaling via PI3K and Akt plays only a minor, conditional role in controlling glucose import into S2 cells.

These results are in marked contrast to the strong dose-dependent inhibition of 2DG import seen following a short (30 minute) pre-treatment with the kinase inhibitor LY294002. This drug inhibits PI3K relatively specifically at low concentrations, but inhibits TOR and other kinases at higher concentrations. LY294002 blocked glucose import nearly completely at either 20 μM or 100 μM (Figure 1G). This indicates that glucose import is in fact regulated in S2 cells, but by an LY294002 target other than PI3K or TOR.

**Figure 2:**
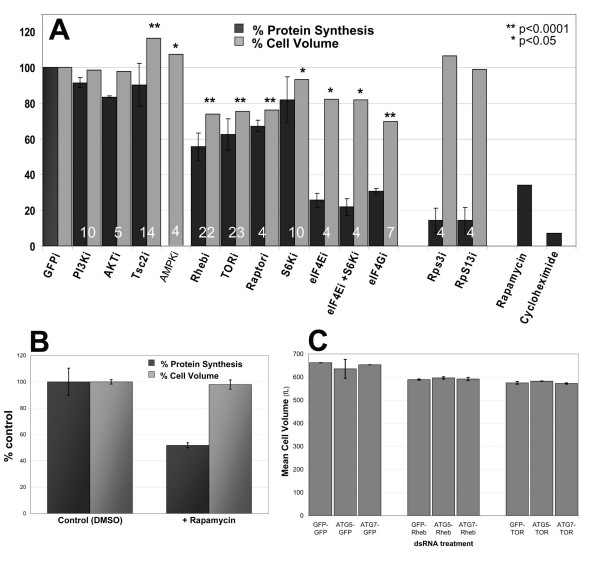
Regulation of translation and cell size by the TOR network. **(A) **Protein synthesis and mean cell volumes are plotted, as percentage of controls. The white number at the base of each pair of bars indicates the number of experiments performed and analyzed for each RNAi treatment. (**B) **Inhibition of TOR blocks protein synthesis before affecting cell size. Cells were pretreated with rapamycin or DMSO (control) for 20 minutes prior to addition of ^3^H-amino acids for a 3-hour translation assay. Translation was markedly reduced in rapamycin-treated cells, while cell size remained normal. (**C) **Co-treatment with dsATG5 or dsATG7 was unable to rescue cell size in dsRheb-treated or dsTOR-treated cells. Cells were pretreated 72 hours with dsATG5, dsATG7, or dsGFP as control to deplete cells of ATG proteins, then the cells were treated with both listed dsRNAs for an additional 72-hour period to determine if loss of autophagy could rescue the cell size. Error bars represent the standard deviations.

To further assess the role of glucose in controlling cell size, we used dsRNA to deplete adenosine monophosphate activated protein kinase (AMPK), a kinase that is activated by AMP when cellular energy levels are low, and which suppresses TOR activity by activating TSC2 [14,42,43]. Depletion of AMPK by RNAi caused an increase in cell size (mean volume for AMPKi = 681fL; GFPi = 636fL, *p *= 0.0011, n = 4; Figure 2A), providing corroborating evidence for a link between cellular energy levels and cell size control that could be mediated by TOR.

### Rheb and TOR are required for normal rates of protein synthesis

Having determined that Rheb and TOR do not drive S2 cell growth by promoting nutrient import, we considered the established role for TOR and its downstream targets in the control of protein synthesis. A translation assay was developed to test whether decreased translation could account for the small size of dsRheb- and dsTOR-treated cells. S2 cells were treated with dsRNA for 72 hours, after which the incorporation of radiolabeled amino acids into protein, relative to total protein, was measured over a 3-hour period. To validate this assay, cells were pretreated for 2 hours with the general protein synthesis inhibitor cycloheximide (100 μg/mL). This caused a reduction in the incorporation of amino acids to approximately 7% of control values. Cells treated with dsRNA directed against Rheb or TOR had a significantly reduced proportion of protein labeled compared to dsGFP-treated controls (approximately 60% of controls) (Figure 2A).

In mammalian and *Drosophila *cells, TOR has been found in two separate complexes; a Rictor-TOR complex, and a Raptor-TOR complex [44-46]. The Raptor-TOR complex appears to be the primary functional complex for serum-dependent or rapamycin-dependent and cell-growth functions of TOR [44]. Accordingly, Raptor was tested for roles in protein synthesis and cell size control. dsRaptor treatment phenocopied dsRheb and dsTOR treatments, in that both cell size and protein synthesis were significantly reduced (Figure 2A). We also found that protein synthesis was markedly reduced, but cell size was unchanged, after a short (20 minute) pre-treatment with 10 ng/mL rapamycin (Figure 2B). Together, these data suggest that a primary effect of Rheb, TOR, and Raptor is to control translation rates.

### Overexpressed Rheb drives protein synthesis in vivo

**Figure 3:**
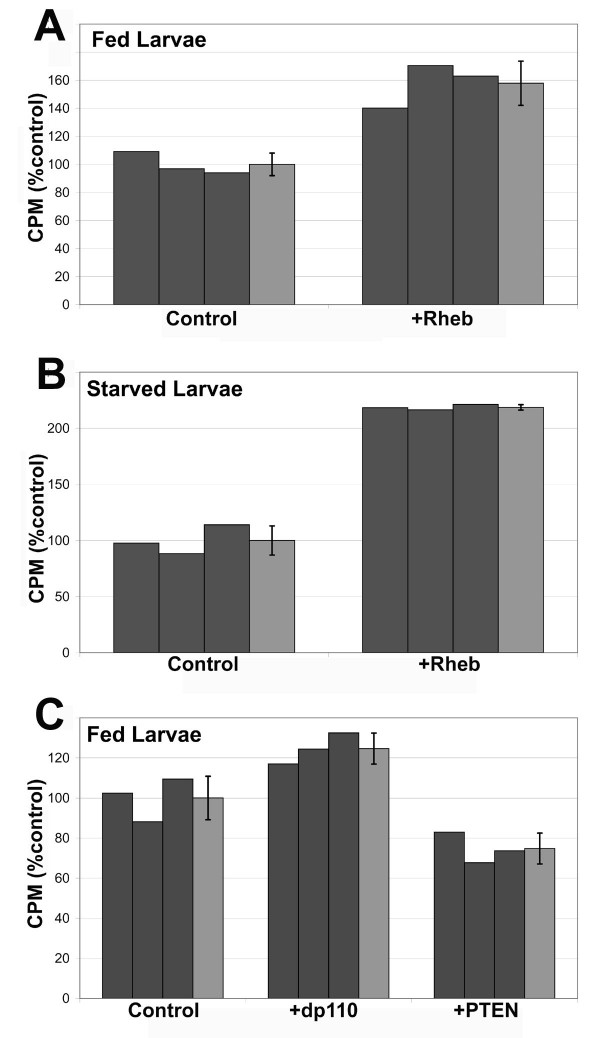
Rheb, PI3K, and PTEN modulate translation in *Drosophila *larvae. Representative experiments in which the indicated gene was overexpressed in whole third instar, pre-wandering larvae using the hsFLP/Gal4-UAS system of induction 20 hrs prior to the translation assay. Gray bars represent means as a percentage of the control mean. **(A) **Rheb overexpression in fed larvae drives protein synthesis. **(B) **Overexpressed Rheb promotes protein synthesis in larvae starved of protein for 32 hrs prior to, and during, induction. **(C) **Dp110, the catalytic subunit of *Drosophila *PI3K, also promotes translation, whereas PTEN, a negative regulator in the insulin/PI3K pathway, inhibits translation. Error bars indicate the standard deviations of the mean.

To ascertain the effects of increasing Rheb activity on protein synthesis, we turned to an *in vivo *assay in *Drosophila *larvae. The *hsFLP/Gal4 *system [47] was used to induce high levels of Rheb expression in most cells of third instar, pre-wandering larvae. The larvae were inverted 20 hours after gene induction, and then incubated for 1 hour in Ringer's saline solution containing ^3^H-amino acids. Protein samples from larval lysates were then prepared and processed as in the S2 cell assay used above. Fed larvae overexpressing Rheb showed an increase in protein synthesis of 140–180% relative to controls (Figure 3A). Previous studies have shown that larvae starved of dietary protein have reduced levels of insulin and TOR signaling activity [33,48,49], and that overexpressed Rheb can drive cell growth in such larvae, which are normally growth-arrested [2]. Thus, we tested whether Rheb was also capable of stimulating protein synthesis in protein-starved larvae. Larvae starved of dietary protein for 32 hours prior to heat-shock induction of Rheb had more than twice the translation rate of controls starved in parallel (Figure 3B). These data indicate that Rheb is a potent regulator of protein synthesis in *Drosophila*, and are consistent with the view that Rheb and TOR are used to modulate rates of protein synthesis in response to changes in the levels of dietary protein.

### Insulin signaling also regulates translation in larvae

Given the abundance of data demonstrating a relationship between TOR activity and insulin signaling, we were surprised to find that suppression of PI3K signaling in S2 cells had no significant effect on cell size or translation (Figure 2A). To further investigate the relationship of insulin signaling to TOR, insulin-signaling components were overexpressed in whole larvae using the hsFLP/Gal4 system. Larvae overexpressing Dp110, the catalytic subunit of PI3K, had a significantly enhanced level of translation relative to controls, though the effect was less profound than that of overexpressed Rheb. Consistently, larvae overexpressing PTEN, a negative regulator of PI3K, had lower levels of protein synthesis than controls (Figure 3C). This result corroborates previous findings that suggest a link between insulin signaling and activation of Rheb-TOR signaling *in vivo*.

### S6K and 4EBP cannot account for the translational effects of TOR

The ribosomal protein S6 kinase (S6K) and eukaryotic initiation factor 4E binding protein (4EBP) are both targets of TOR in mammals and flies [50] and are well-known regulators of protein synthesis. Flies and mice mutant for s*6k *have reduced body size due to a reduction in cell size, although they have a normal number of total body cells [30,51]. Treatment of S2 cells with dsRNA directed against S6K also reduced cell size and protein synthetic rate, but not as drastically as did dsRheb or dsTOR treatment (Figure 2A). This is consistent with our previous finding that overexpressed Rheb can drive cell growth in *s6k *mutant animals [2], and indicates that S6K is not the sole mediator of cell size and protein synthesis downstream of TOR.

4EBP is a well-characterized TOR target that functions by sequestering the mRNA cap-binding protein, eukaryotic initiation factor 4E (eIF4E). Phosphorylation of 4EBP by TOR releases eIF4E and allows it to bind the eIF4G scaffold protein, which, in a complex with other components, binds the 5' cap region of mRNAs to initiate translation. Ectopic expression of an active form of *Drosophila *4EBP (known as Thor), a negative regulator of translation, results in reduced wing size due to reductions in cell size and cell number [52]. Given these observations, we attempted to rescue the cell size and translation defects observed in dsTOR-treated S2 cells by codepleting 4EBP with dsRNA. Treatment with ds4EBP alone had no detectable effect on cell size or protein synthesis rates. Moreover, in co-depletion experiments, ds4EBP did not lessen the reductions in cell size and translation caused by dsTOR or dsRheb treatment (data not shown). Therefore, negative regulation of 4EBP by Rheb and TOR appears not to be an important mode of growth control in S2 cells. We did find, however, that dsRNA directed against *Drosophila *eIF4E or eIF4G resulted in small cells with greatly reduced translation (Figure 2A). Considering the similarity to the dsTOR and dsRheb phenotypes, this is consistent with the proposal that TOR is required for proper eIF4G function and translation initiation [53].

### Small cell size is a TOR pathway-specific phenotype

Because inhibition of any component of the protein synthesis machinery could be reasoned to have these same phenotypic consequences, two ribosomal proteins were also tested. Treatment of cells with dsRNA targeting ribosomal proteins S3 or S13 did not cause a demonstrable alteration in cell size, despite severely reducing rates of translation (Figure 2A) and cell division (data not shown). Although this failure to affect cell size could be due to differences in the kinetics of target depletion by different types of RNAi, we believe it is meaningful because, whereas the TOR pathway suppression invariably reduces cell size, reducing protein synthesis by other means rarely does so. For instance, heterozygosity for ribosomal gene mutations (*Minutes*) slows growth dramatically *in vivo*, yet does not reduce cell size, and sometimes even increases it (our unpublished observations). Moreover, whole-genome screening using RNAi in S2 cells has failed to detect ribosomal protein genes as regulators of cell size [54], whereas TOR pathway components and targets are readily detected [31,54]. Hence, we surmise that smaller cell size is not a necessary consequence of reduced protein synthesis, and that other targets in addition to S6K and 4EBP mediate the effects of TOR on cell size. A recent report duplicating many of these results corroborates these conclusions [31].

### Autophagy

Recent studies have demonstrated that TOR activity suppresses autophagy, a catabolic process whereby starved cells engulf and break down their cytoplasm and organelles, which occurs in flies, yeast, and mammalian cells [9,55,56]. We therefore considered the possibility that depletion of TOR in S2 cells may induce autophagy, and that this might account for the reduced cell size seen in dsTOR-treated or dsRheb-treated cells. To test this hypothesis, two proteins required for autophagy, ATG5 and ATG7 [9], were targeted for depletion using RNAi, either alone or in combination with dsTOR or dsRheb treatment. In some cases, we pre-depleted the cells of ATG5 or ATG7 by applying the RNAi against these targets 3days prior to RNAi directed against Rheb or TOR. In all cases, we found that depletion of these ATG mRNAs had no significant effect on cell size, and did not rescue the reduction in cell size caused by depletion of either Rheb or TOR (Figure 2C). These results support the hypothesis that the reduction in cell size is primarily due to the reduction in protein synthesis, and not to an increase in protein breakdown via autophagy.

### TOR and Rheb promote ribosome biogenesis

**Figure 4:**
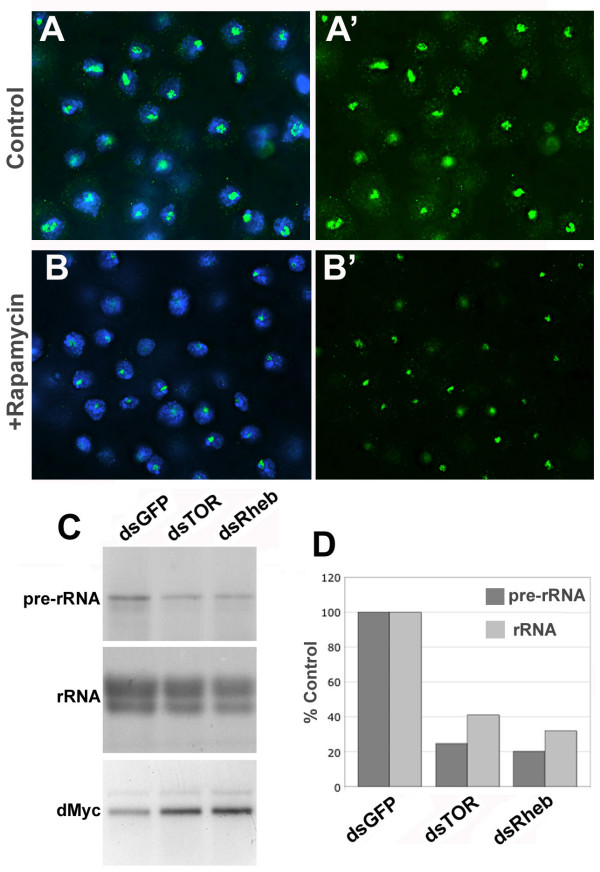
**Rheb and TOR regulate ribosome biogenesis**. **(A, B) **Nucleoli visualized by anti-fibrillarin staining (green) [84], in S2 cells treated with **(A) **DMSO or **(B) **rapamycin for 16h. DNA was visualized using Hoechst 33258 (blue). **(C) **Northern blots of RNA from S2 cells treated for 72h with the indicated dsRNA. A probe for an internal transcribed spacer (ITS) region of the rDNA was used to detect pre-rRNA. Total rRNA was detected with ethidium bromide. A probe for dMyc transcript was used as a loading control. **(D) **Quantification, using ImageJ software, of pre-rRNA and total rRNA, relative to dMyc mRNA, from the samples shown in (C).

Given that S6K, 4EBP, and autophagy appeared to be insufficient to account for the effects of Rheb and TOR on protein synthesis and cell size, we sought other explanations. Because TOR signaling regulates ribosome biogenesis in both yeast and mammalian cells [7,8], the effect of loss of either Rheb or TOR on ribosome synthesis in S2 cells was examined. S2 cells treated with dsRheb and dsTOR showed reduced levels of rRNA synthesis as measured by Northern blotting using an internal transcribed spacer probe that hybridizes to the 45S pre-rRNA (Figure 4C). Similarly, rapamycin-treated S2 cells also had reduced levels of pre-rRNA (data not shown). Levels of total (mature) rRNA were also reduced in both dsRheb-treated and dsTOR-treated cells, indicating that these cells have fewer ribosomes (Figure 4C). Consistent with this result, S2 cells treated with rapamycin had smaller nucleoli than controls (Figures 4A, B). In contrast, depletion of either PI3K or Akt had no detectable effect on either pre-rRNA or total rRNA levels in S2 cells (data not shown). This is consistent with our finding that neither dsPI3K nor dsAkt treatment significantly affected either translation nor cell size in our S2 cell assays. Thus, Rheb and TOR regulate ribosome biogenesis in *Drosophila *S2 cells, and do so independently of PI3K and Akt.

## Discussion

We initiated this study to determine which of the downstream outputs of the insulin-TOR signaling network are the most important effectors of cell growth, and also to probe the functional relationships between Rheb, TOR, and the insulin signaling system. The literature reports many growth-associated processes as being dependent upon insulin signaling and/or TOR function [10,25]. These include translational initiation [6,57], ribosome biogenesis [7,8], glucose import and glycolysis [5,24], amino-acid import [5], autophagy [55], transcriptional programs relevant to metabolism and stress response [38,58-60], and control of the actin cytoskeleton [61]. While some of these processes are affected directly by Akt-dependent or TOR-dependent phosphorylation of known substrates, others probably represent indirect effects of general changes in metabolism caused by altering insulin or TOR signaling. As the relative importance of different growth processes will vary according to cell type and organism, comparisons of data from different experimental systems are often inconclusive. Hence we surveyed a number of growth-related processes in a single cell type, the *Drosophila *S2 cell. These embryo-derived cells are thought to be related to phagocytic haematocytes. They are relatively well characterized and are highly sensitive to RNAi-mediated suppression of gene function. TOR activity in S2 cells, as assayed by S6K or 4EBP phosphorylation, has been demonstrated to be responsive to insulin signaling and to glucose and amino acid levels, at least in short-term assays [2,40,41,49,50].

First, we noted that depletion of Rheb, TOR, or Raptor, or treatment of cells with rapamycin, caused very similar effects on all the growth-related processes that we assayed. This is in agreement with the prevailing view in which these three factors function together as a regulatory module [26,27,44,62]. Second, we found that the depletion of Rheb or TOR decreased rates of bulk protein synthesis and ribosome production without reducing rates of bulk amino acid import, arginine import, or glucose import. This implies that Rheb does not regulate TOR by controlling nutrient availability (as we suggested in an earlier publication [2]) but is consistent with the proposal that Rheb activates TOR by direct physical interaction [26-29]. Our results also imply that Rheb/TOR signaling does not control cell growth via direct effects on nutrient import. One caveat to this interpretation is that the reduction in growth rate resulting from depletion of Rheb or TOR is small, amounting to a ~50% reduction in the mass of an S2 cell culture over 72 hrs. Even considering that most of this reduction in growth is sustained during the last 24 hrs of culture, after the RNAi becomes fully penetrant, it is doubtful that we could detect the small deficits in nutrient import (< 5%) required to explain this mass deficit using assays lasting at most 1 hr. Notwithstanding this caveat, the effects of TOR inhibition on protein synthesis were large and rapid and preceded decreases in cell size (Figure 2B), suggesting that the loss of cell mass after TOR inhibition is likely to be due to the reduction in protein synthesis.

Other factors could of course contribute to the effects of Rheb/TOR signaling on cell size and growth rate. These include changes in the relative rates of nutrient storage, utilization for energy, export, and of macromolecule degradation and cell death. The balance of all these processes determines the steady-state cell size and growth rate of the culture as a whole. Although we did not assay all of these processes after suppressing TOR function, we did attempt to rescue the TOR-dependent reduction in cell size by inhibiting autophagy, using RNAi directed at two genes required for that process. No rescue was achieved, suggesting that the loss of S2 cell mass that occurs after Rheb or TOR inhibition is not due to increased autophagy. Studies performed *in vivo *in *Drosophila *larvae, using a similar strategy, have arrived at the same conclusion [9].

Although we favor the idea that the cell size reduction that occurs when TOR activity is suppressed results from reduced protein synthesis, we note that these cell-size effects were specific to TOR pathway components, and were not reproduced using RNAi directed against ribosomal proteins, or by the translation inhibitor cycloheximide (Figure 2). This specificity is also observed *in vivo*, where mutations in ribosomal protein genes (*Minutes*) reduce growth rates, but do not reduce cell size. The origin of the TOR-specific cell size decrease remains a mystery, but may lie in the differential effects of TOR on the translation of specific classes of mRNAs, such as cap-dependent, internal ribosome entry site-dependent, and 5' terminal oligo-pyrimidine-containing mRNAs. By having relatively severe effects on the translation of mRNAs encoding products involved in processes such as protein turnover, nutrient storage, or cell cycle progression, TOR might alter the balance between rates of cell growth and division, and thus affect cell size. Analysis of the effects of TOR on the translation of the entire spectrum of specific proteins may clarify how it controls cell size. As noted above, it is also possible that TOR-mediated regulation of growth-related processes unrelated to protein synthesis, such as transcription or nutrient utilization, account for much of TOR's effect on cell size (see, for instance Guertin et al [31]).

TOR has several well-characterized targets that modulate protein synthesis, notably the ribosomal protein S6K and the initiation factor 4EBP (reviewed by Hay and Sonenberg [6] and Wullschleger et al[10]). Our data indicate that S6K and the 4EBP target eIF4E, which can be activated by TOR, could account for a portion of the effect of TOR on protein synthesis and cell size in S2 cells, although not the total effect (Figure 2A). This is consistent with *in vivo *studies in *Drosophila *and mice showing that S6K, in contrast to Rheb and TOR, is a non-essential gene product that has relatively mild effects on cell growth in normal culture conditions [30,51], and that 4EBP is dispensable [52]. Thus, many results underline the importance of identifying factors other than S6K and 4EBP, through which Rheb/TOR signaling regulates protein synthesis. The literature suggests that TOR regulates protein synthesis through multiple targets that work both directly, by controlling rates of translation initiation and elongation, and indirectly, by controlling ribosome supply. TOR may control ribosome supply in part via TIF-IA and UBF, two regulators of RNA polymerase I activity [63,64]. In yeast, TOR also regulates the expression of ribosomal protein gene expression via protein kinase A (PKA) and the forkhead-like transcription factor, FHL1 [7,65]. However, his mechanism is not supported by gene expression profiling experiments in S2 cells [31], which show that TOR regulates many genes involved in ribosome assembly, but does not affect the expression of mRNAs encoding the ribosomal proteins. Interestingly, RNAi-mediated suppression of 54 TOR-regulated genes (65% of the TOR targets identified) reduced cell size, suggesting that the total effect of TOR on cell size is the sum of many small transcriptional effects. Finally, we note that experiments with rapamycin show that TOR has immediate effects on protein synthesis that are unlikely to result from reduced ribosome numbers. In addition to S6K and 4EBP, these rapid effects could result from the effects of TOR on the elongation factor eEF2 [66] or on the translation initiation factors eIF2α [67] and eIF4G [53].

Another issue addressed here is the relationship of insulin signaling to TOR activity. Many studies have implicated insulin signaling as an important regulator of TOR activity [12,41,50,68-70], and two general mechanisms have been proposed to explain this control. The first is a direct mechanism in which TSC2 is inactivated by phosphorylation by Akt [71-74]. The second is an indirect mechanism in which insulin signaling promotes glucose import and raises the ATP/AMP ratio, which suppresses AMPK and thereby inactivates TSC2 [5,14,39,42,43]. While both are attractive mechanisms of control, our data suggests that they play relatively minor roles in controlling TOR in S2 cell culture. We found that inhibition of PI3K or Akt with either RNAi or chemical inhibitors had much smaller effects on cell size and protein synthesis than did inhibition of TOR, Rheb, or Raptor (Figure 2). Three independent studies have corroborated our observations regarding cell size [31,54,75]. Furthermore, we found that insulin and some of its downstream effectors are not required for glucose import in S2 cells, and that insulin significantly stimulated glucose import only when the cells had been serum-starved for a short time (Figure 1G). Consistent with these observations, we and others have found that S2 cells can grow and proliferate in serum-free media without insulin [76]. Along with other studies [49,50,69,74], these results indicate that insulin signaling via PI3K and Akt does not act as an important regulator of TOR activity in all cellular contexts. This is likely to be the case in S2 cells, at least when they are grown in nutrient-rich media. TOR activity has also been found to be insulin-independent in mammalian CHO and HEK293 cells [77], and so this relationship may not be so unusual.

Considering the limited affects of insulin, PI3K, and Akt on S2 cell growth, the rapid TOR-dependent phosphorylation of S6K and 4EBP that occurs when these cells are stimulated with insulin [41,50,69] seems paradoxical. Although we cannot explain this apparent discrepancy, we suggest that insulin stimulation may have transient effects (e.g. S6K phosphorylation) that do not correlate well with long-term outcomes such as changes in cell size or ribosome production. It may also be that the "basal" insulin-independent level of TOR activity in S2 cells grown in nutrient-rich media is sufficient to maintain high rates of protein synthesis and a large cell size. Further activation of TOR in this context, by insulin stimulation or loss of TSC2, might increase S6K and 4EBP phosphorylation without appreciably upregulating actual cell growth. This explanation seems consistent with previously published data, which documented insulin-dependent phosphorylation of TOR targets in S2 cells cultured under the same conditions we used, but did not assay the effects of insulin signaling on cell growth. Our observation that depletion of TSC2 in S2 cells did not detectably increase protein synthesis rates, and caused only a modest increase in cell size (Figure 2), is also consistent with this explanation. In other cellular contexts, such as in feeding larvae or adult flies, insulin signaling does have profound effects on glucose metabolism [37,38], cell growth, and size [48,78-81]. Thus, we believe that insulin signaling is a major regulator of both cellular energy levels and TOR activity in many contexts *in vivo*. Indeed, we were able to document a significant effect of insulin signaling on protein synthesis in living *Drosophila *larvae (Figure 3C). Even *in vivo *however, recent evidence suggests that the requirement for insulin signaling may vary according to tissue type [37].

## Conclusion

In summary, our results argue against a significant or direct role for Rheb-TOR signaling in promoting nutrient import, but corroborate the established role of TOR as an important regulator of protein synthesis and ribosome production. Our findings also highlight the variable relationship between insulin signaling and TOR activity, and cast doubt upon the relevance of insulin as a regulator of TOR in *Drosophila *S2 cells.

## Methods

### dsRNA synthesis

cDNAs encoding the proteins of interest were obtained and amplified by PCR using composite primers (details available on request), of which the 3' 20 base pairs were gene-specific and the 5' ends had a T7 RNA polymerase binding site. The double-stranded PCR products, which contained a portion of the coding sequence for the protein to be depleted flanked by T7 polymerase binding sites, were purified using a commercial kit (QIAquick Gel Extraction kit; Qiagen, Valencia CA, USA) and then used as template for RNA synthesis using a T7 RNA polymerase (Megascript T7 kit; Ambion, Austin TX, USA). RNA was precipitated by standard procedures using sodium acetate and ethanol for precipitation. To anneal complementary transcribed products, the RNA was heated to 65°C for 30 minutes and slowly cooled to room temperature before being quantified by UV spectrophotometry and diluted to a working concentration of 1 μg/μL. Template DNA for T7-cDNA synthesis was obtained from the DGC clone collection or by reverse transcription (SuperScript II; Invitrogen, Carlsbad, CA, USA) and PCR amplification of an S2 RNA product using sequence-specific primers. In the latter cases, the T7-cDNA was sequenced to verify that the expected product was obtained. The *Drosophila *genome encodes seven highly related eIF4E genes [82], and the dsRNA we used is predicted to target the four most abundantly expressed isoforms.

### dsRNA interference

Interference was performed as described previously [83]. Briefly, S2 cells from an actively growing cell culture were collected by centrifugation and resuspended in serum-free media (SFM, Hy-Q, Hy-Clone, Logan UT, USA) at a density of 1 × 10^6 ^cells/mL in one well of a six-well plate, then 20 μg dsRNA was added to the cells. After 1 hour of incubation in the SFM, 2 mL Schneider's *Drosophila *medium containing 10% fetal bovine serum (Invitrogen/Gibco, Carlsbad, CA, USA) (SDM-FBS) was added to each well. After 72 hours of incubation at 25°C to allow for the turnover of the protein of interest, cells were collected for use in the assays.

### Tritiated-nutrient import assays

Cells were pelleted and resuspended to a density of about 1.5 million cells/mL in uptake assay buffer (UAB; 5.4 mM KCl, 140 mM NaCl, 1.8 mM CaCl_2_, 0.8 mM MgSO_4_, 25 mM Hepes, 25 mM Tris), pH 7.5 [5] containing 10 μg/mL bovine insulin (Sigma), and if used, inhibitors such as LY294002 (Calbiochem, San Diego, CA, USA), AKT inhibitor (1L-6-Hydroxymethyl-chiro-inositol 2-(R)-2-O-methyl-3-O-octadecylcarbonate; Calbiochem) or rapamycin (LC Laboratories, Wobern MA, USA). After 30 minutes equilibration time, 10 μCi/mL tritiated nutrient (15 ^3^H-amino acid mix; 16–92 Ci/mmol; TRK-440), ^3^H-arginine (60 Ci/mmol; TRK698), or 2DG (Amersham Biosciences, Piscataway NJ, USA) was added to each cell suspension. The suspension was mixed by inversion. Assays were carried out at room temperature over a period of 45–90 minutes. Duplicate aliquots of each cell suspension were removed at 15–20 minute intervals for the duration of each assay. Aliquots were collected and washed twice with UAB to remove any extracellular tritiated nutrient. The cells were then lysed with cell lysis buffer (100 mM Tris-Cl, pH 8.0, 100 mM NaCl, 0.5% Triton X-100), vortexed, and incubated in ice for at least 10 minutes to ensure complete lysis. Lysates were transferred to scintillation vials containing 3 mL scintillation buffer (EcoScint Original; National Diagnostics, Atlanta GA, USA). The vials were capped and mixed by inversion before the activity count was obtained using a scintillation counter (Beckman LS6500, Beckman-Coulter, Fullerton CA, USA). The activity (counts per minute; cpm) for each time point analyzed was graphically illustrated using MS Excel for visual comparison of nutrient uptake kinetics of the control versus experimental cell populations. If two separate populations were used for control and experimental groups, as was the case with dsRNA-treated cells, the activity was normalized to the number of cells/mL, cell volume/cell/mL, or protein content. Experiments were carried out a minimum of three times.

### Determination of cell size

The mean cell size for each culture was determined using a counter (Beckman Coulter Z2) set to count cells of size 5–20 μm. dsRNA-treated cultures were compared to paired control dsGFP-treated cultures.

### Determination of protein content

Protein content of a cell culture was determined using the DC microplate protein assay (Biorad). Cell lysis was carried out using the cell lysis buffer as described above.

### Translation assay, S2 cells

In total, 2 mL of S2 cells in cultured medium was transferred to one well of a six-well dish, then 1 mL of fresh SDM-FBS containing 15 μCi/mL tritiated amino acid mix (Amersham Biosciences) was added to each well for a final concentration of 5 μCi AA per mL culture. Following 3 hours of amino-acid incorporation at room temperature, 900 μL of suspended cells were split into three aliquots, pelleted, and the media removed. The cells were washed once with 200 μL UAB to remove excess non-imported amino acids. The cells were lysed in 150 μL cell lysis buffer as above then 12 μL resin (Strataclean; Stratagene) was added to each vial of cell lysate. The vials were vortexed to disperse the resin and then left for at least 3 minutes to allow the resin to bind the protein. The resin was pelleted by centrifugation and the remaining lysate was decanted. The resin was washed once with 100 μL UAB before being transferred to scintillation vials containing 3 mL scintillation liquid (EcoScint Original; National Diagnostics). Using a scintillation counter (Beckman LS6500) 10-minute scintillation counts were performed. To validate the assay conditions, a resin-binding test was performed. Radioactivity of resin-bound material (protein), as measured in cpm, was linear over the range of 5–20 mL resin volume. The resin did not bind native amino acids. Therefore, using a constant resin volume normalizes for protein content in this assay. Incorporation of ^3^H-amino acids into resin-binding material (protein) was also linear over a range of 25–350 minutes incorporation time.

### Translation assay, whole larvae

The S2 translation protocol above was modified for use with whole larvae. Experimental larval genotypes were: *hs-Flp; Act>CD2>Gal4 UAS-GFP/UAS-Rheb *(Figures 3A, B), and *hs-Flp; Act>CD2>Gal4 UAS-GFP/UAS-Dp110 *or *hs-Flp; Act>CD2>Gal4 UAS-GFP/UAS-PTEN *(Figure 3C). Control genotypes were: *hs-Flp; Act>CD2>Gal4 UAS-GFP *in each case. Triplicate sets of 10 larvae were inverted in Ringer's solution and transferred to an eppendorf tube along with 100 μL Ringer's solution for the assay; 750 μL Ringer's solution containing 15 μCi/mL tritiated amino acid mix was added to each eppendorf tube. The larval carcasses were rocked at room temperature for 1 hour, washed in 500 μL cold Ringer's solution, and lysed using mechanical disruption of the larval carcasses in a total of 350 μL cell-lysis buffer. Larval debris was pelleted, and 250 μL lysate was transferred to a new vial. A quantity (15 μL) of resin (Strataclean) resin was added to the lysate. Protein-bound resin was then pelleted, washed with 150 μL Ringer's solution and transferred to 3 mL scintillation buffer, and 1-minute counts were obtained (Beckman LS6500).

### Northern blotting

A quantity (1 mL) of dsRNA-treated S2 cell culture (~2 million cells) was used for RNA extraction. Total RNA was extracted using 1 mL of reagent (TRIzol; Invitrogen) using standard procedures, followed by DNase I treatment for 15 minutes at 37°C and purification of the RNA (RNEasy kit; Qiagen). RNA was stored at -70°C until it could be used for Northern blot analysis. Equal volumes of total RNA were loaded into a MOPS/formaldehyde gel for electrophoretic separation. The RNA was blotted onto a positively charged nylon membrane (Roche) according to standard procedures, and the transcript of interest was detected using DIG-labeled antisense probes (DIG labeling and detection, Roche).

## Authors' contributions

DJH performed the RNAi, nutrient import and protein synthesis assays, detailed in Figs 1, 2, 3. SSG performed certain aspects of the assays detailed in Figure 4. AFAdlC performed the Arg uptake experiments in Figure 1. BAE conceived of the study, participated in its design and coordination, and helped to draft and edit the manuscript. All authors read and approved of the final manuscript.

## Supplementary Material

Additional file 1 Supplementary Figure 1: RNAi treatment depletes mRNAs encoding components of the insulin/TOR signaling pathway. We show representative northern blots hybridized with RNA probes directed at the indicated targets. RNA was harvested from equal numbers of S2 cells treated with a control RNAi (GFP RNAi) or a target specific RNAi (indicated), for three days. Click here for file

## References

[B1] Patel PH, Thapar N, Guo L, Martinez M, Maris J, Gau CL, Lengyel JA, Tamanoi F (2003). Drosophila Rheb GTPase is required for cell cycle progression and cell growth. J Cell Sci.

[B2] Saucedo LJ, Gao X, Chiarelli DA, Li L, Pan D, Edgar BA (2003). Rheb promotes cell growth as a component of the insulin/TOR signalling network. Nat Cell Biol.

[B3] Stocker H, Radimerski T, Schindelholz B, Wittwer F, Belawat P, Daram P, Breuer S, Thomas G, Hafen E (2003). Rheb is an essential regulator of S6K in controlling cell growth in Drosophila. Nat Cell Biol.

[B4] Avruch J, Lin Y, Long X, Murthy S, Ortiz-Vega S (2005). Recent advances in the regulation of the TOR pathway by insulin and nutrients. Curr Opin Clin Nutr Metab Care.

[B5] Edinger AL, Thompson CB (2002). Akt maintains cell size and survival by increasing mTOR-dependent nutrient uptake. Mol Biol Cell.

[B6] Hay N, Sonenberg N (2004). Upstream and downstream of mTOR. Genes Dev.

[B7] Martin DE, Soulard A, Hall MN (2004). TOR regulates ribosomal protein gene expression via PKA and the Forkhead transcription factor FHL1. Cell.

[B8] Powers T, Walter P (1999). Regulation of ribosome biogenesis by the rapamycin-sensitive TOR-signaling pathway in Saccharomyces cerevisiae. Mol Biol Cell.

[B9] Scott RC, Schuldiner O, Neufeld TP (2004). Role and regulation of starvation-induced autophagy in the Drosophila fat body. Dev Cell.

[B10] Wullschleger S, Loewith R, Hall MN (2006). TOR signaling in growth and metabolism. Cell.

[B11] Castro AF, Rebhun JF, Clark GJ, Quilliam LA (2003). Rheb binds tuberous sclerosis complex 2 (TSC2) and promotes S6 kinase activation in a rapamycin- and farnesylation-dependent manner. J Biol Chem.

[B12] Garami A, Zwartkruis FJ, Nobukuni T, Joaquin M, Roccio M, Stocker H, Kozma SC, Hafen E, Bos JL, Thomas G (2003). Insulin activation of Rheb, a mediator of mTOR/S6K/4E-BP signaling, is inhibited by TSC1 and 2. Mol Cell.

[B13] Inoki K, Li Y, Xu T, Guan KL (2003). Rheb GTPase is a direct target of TSC2 GAP activity and regulates mTOR signaling. Genes Dev.

[B14] Inoki K, Zhu T, Guan KL (2003). TSC2 mediates cellular energy response to control cell growth and survival. Cell.

[B15] Li Y, Inoki K, Guan KL (2004). Biochemical and functional characterizations of small GTPase Rheb and TSC2 GAP activity. Mol Cell Biol.

[B16] Tabancay AP, Gau CL, Machado IM, Uhlmann EJ, Gutmann DH, Guo L, Tamanoi F (2003). Identification of dominant negative mutants of Rheb GTPase and their use to implicate the involvement of human Rheb in the activation of p70S6K. J Biol Chem.

[B17] Tee AR, Manning BD, Roux PP, Cantley LC, Blenis J (2003). Tuberous sclerosis complex gene products, Tuberin and Hamartin, control mTOR signaling by acting as a GTPase-activating protein complex toward Rheb. Curr Biol.

[B18] Zhang Y, Gao X, Saucedo LJ, Ru B, Edgar BA, Pan D (2003). Rheb is a direct target of the tuberous sclerosis tumour suppressor proteins. Nat Cell Biol.

[B19] Beugnet A, Tee AR, Taylor PM, Proud CG (2003). Regulation of targets of mTOR (mammalian target of rapamycin) signalling by intracellular amino acid availability. Biochem J.

[B20] Hara K, Yonezawa K, Weng QP, Kozlowski MT, Belham C, Avruch J (1998). Amino acid sufficiency and mTOR regulate p70 S6 kinase and eIF-4E BP1 through a common effector mechanism. J Biol Chem.

[B21] Kessler A, Tomas E, Immler D, Meyer HE, Zorzano A, Eckel J (2000). Rab11 is associated with GLUT4-containing vesicles and redistributes in response to insulin. Diabetologia.

[B22] Sano H, Kane S, Sano E, Miinea CP, Asara JM, Lane WS, Garner CW, Lienhard GE (2003). Insulin-stimulated phosphorylation of a Rab GTPase-activating protein regulates GLUT4 translocation. J Biol Chem.

[B23] Yang W, Tabancay AP, Urano J, Tamanoi F (2001). Failure to farnesylate Rheb protein contributes to the enrichment of G0/G1 phase cells in the Schizosaccharomyces pombe farnesyltransferase mutant. Mol Microbiol.

[B24] Koumanov F, Jin B, Yang J, Holman GD (2005). Insulin signaling meets vesicle traffic of GLUT4 at a plasma-membrane-activated fusion step. Cell Metab.

[B25] Crespo JL, Hall MN (2002). Elucidating TOR signaling and rapamycin action: lessons from Saccharomyces cerevisiae. Microbiol Mol Biol Rev.

[B26] Long X, Lin Y, Ortiz-Vega S, Yonezawa K, Avruch J (2005). Rheb binds and regulates the mTOR kinase. Curr Biol.

[B27] Long X, Ortiz-Vega S, Lin Y, Avruch J (2005). Rheb binding to mammalian target of rapamycin (mTOR) is regulated by amino acid sufficiency. J Biol Chem.

[B28] Urano J, Comiso MJ, Guo L, Aspuria PJ, Deniskin R, Tabancay AP, Kato-Stankiewicz J, Tamanoi F (2005). Identification of novel single amino acid changes that result in hyperactivation of the unique GTPase, Rheb, in fission yeast. Mol Microbiol.

[B29] Smith EM, Finn SG, Tee AR, Browne GJ, Proud CG (2005). The tuberous sclerosis protein TSC2 is not required for the regulation of the mammalian target of rapamycin by amino acids and certain cellular stresses. J Biol Chem.

[B30] Montagne J, Stewart MJ, Stocker H, Hafen E, Kozma SC, Thomas G (1999). *Drosophila *S6 kinase: a regulator of cell size. Science.

[B31] Guertin DA, Guntur KV, Bell GW, Thoreen CC, Sabatini DM (2006). Functional Genomics Identifies TOR-Regulated Genes that Control Growth and Division. Curr Biol.

[B32] Hennig KM, Colombani J, Neufeld TP (2006). TOR coordinates bulk and targeted endocytosis in the Drosophila melanogaster fat body to regulate cell growth. J Cell Biol.

[B33] Colombani J, Raisin S, Pantalacci S, Radimerski T, Montagne J, Leopold P (2003). A nutrient sensor mechanism controls Drosophila growth. Cell.

[B34] Urano J, Tabancay AP, Yang W, Tamanoi F (2000). The Saccharomyces cerevisiae Rheb G-protein is involved in regulating canavanine resistance and arginine uptake. Journal of Biological Chemistry.

[B35] Zeigerer A, Lampson MA, Karylowski O, Sabatini DD, Adesnik M, Ren M, McGraw TE (2002). GLUT4 retention in adipocytes requires two intracellular insulin-regulated transport steps. Mol Biol Cell.

[B36] Wick AN, Drury DR, Nakada HI, Wolfe JB (1957). Localization of the primary metabolic block produced by 2-deoxyglucose. J Biol Chem.

[B37] Shingleton AW, Das J, Vinicius L, Stern DL (2005). The temporal requirements for insulin signaling during development in Drosophila. PLoS Biol.

[B38] Rulifson EJ, Kim SK, Nusse R (2002). Ablation of insulin-producing neurons in flies: growth and diabetic phenotypes. Science.

[B39] Hahn-Windgassen A, Nogueira V, Chen CC, Skeen JE, Sonenberg N, Hay N (2005). Akt Activates the Mammalian Target of Rapamycin by Regulating Cellular ATP Level and AMPK Activity. J Biol Chem.

[B40] Radimerski T, Montagne J, Rintelen F, Stocker H, Van der Kaay J, Downes CP, Hafen E, Thomas G (2001). dS6K Regulated Cell Growth in dPKB/dPI3K Independent, but Requires dPDK1.

[B41] Lizcano JM, Alrubaie S, Kieloch A, Deak M, Leevers SJ, Alessi DR (2003). Insulin-induced Drosophila S6 kinase activation requires phosphoinositide 3-kinase and protein kinase B. Biochem J.

[B42] Corradetti MN, Inoki K, Bardeesy N, DePinho RA, Guan KL (2004). Regulation of the TSC pathway by LKB1: evidence of a molecular link between tuberous sclerosis complex and Peutz-Jeghers syndrome. Genes Dev.

[B43] Shaw RJ, Bardeesy N, Manning BD, Lopez L, Kosmatka M, DePinho RA, Cantley LC (2004). The LKB1 tumor suppressor negatively regulates mTOR signaling. Cancer Cell.

[B44] Kim DH, Sarbassov DD, Ali SM, King JE, Latek RR, Erdjument-Bromage H, Tempst P, Sabatini DM (2002). mTOR interacts with raptor to form a nutrient-sensitive complex that signals to the cell growth machinery. Cell.

[B45] Sarbassov DD, Guertin DA, Ali SM, Sabatini DM (2005). Phosphorylation and regulation of Akt/PKB by the rictor-mTOR complex. Science.

[B46] Yonezawa K, Tokunaga C, Oshiro N, Yoshino K (2004). Raptor, a binding partner of target of rapamycin. Biochem Biophys Res Commun.

[B47] Pignoni F, Zipursky S (1997). Induction of *Drosophila *eye development by decapentalegic. Development.

[B48] Britton JS, Lockwood WK, Li L, Cohen SM, Edgar BA (2002). Drosophila's insulin/PI3-kinase pathway coordinates cellular metabolism with nutritional conditions. Developmental Cell.

[B49] Oldham S, Montagne J, Radimerski T, Thomas G, Hafen E (2000). Genetic and biochemical characterization of dTOR, the Drosophila homolog of the target of rapamycin. Genes & Development.

[B50] Miron M, Lasko P, Sonenberg N (2003). Signaling from Akt to FRAP/TOR targets both 4E-BP and S6K in Drosophila melanogaster. Mol Cell Biol.

[B51] Pende M, Um SH, Mieulet V, Sticker M, Goss VL, Mestan J, Mueller M, Fumagalli S, Kozma SC, Thomas G (2004). S6K1(-/-)/S6K2(-/-) mice exhibit perinatal lethality and rapamycin-sensitive 5'-terminal oligopyrimidine mRNA translation and reveal a mitogen-activated protein kinase-dependent S6 kinase pathway. Mol Cell Biol.

[B52] Miron M, Verdu J, Lachance PE, Birnbaum MJ, Lasko PF, Sonenberg N (2001). The translational inhibitor 4E-BP is an effector of PI(3)K/Akt signalling and cell growth in Drosophila. Nature Cell Biology.

[B53] Raught B, Gingras AC, Gygi SP, Imataka H, Morino S, Gradi A, Aebersold R, Sonenberg N (2000). Serum-stimulated, rapamycin-sensitive phosphorylation sites in the eukaryotic translation initiation factor 4GI. Embo J.

[B54] Bjorklund M, Taipale M, Varjosalo M, Saharinen J, Lahdenpera J, Taipale J (2006). Identification of pathways regulating cell size and cell-cycle progression by RNAi. Nature.

[B55] Klionsky DJ (2005). The molecular machinery of autophagy: unanswered questions. J Cell Sci.

[B56] Rusten TE, Lindmo K, Juhasz G, Sass M, Seglen PO, Brech A, Stenmark H (2004). Programmed autophagy in the Drosophila fat body is induced by ecdysone through regulation of the PI3K pathway. Dev Cell.

[B57] Gingras AC, Raught B, Sonenberg N (2001). Regulation of translation initiation by FRAP/mTOR. Genes Dev.

[B58] Broughton SJ, Piper MD, Ikeya T, Bass TM, Jacobson J, Driege Y, Martinez P, Hafen E, Withers DJ, Leevers SJ (2005). Longer lifespan, altered metabolism, and stress resistance in Drosophila from ablation of cells making insulin-like ligands. Proc Natl Acad Sci USA.

[B59] Teleman AA, Chen YW, Cohen SM (2005). 4E-BP functions as a metabolic brake used under stress conditions but not during normal growth. Genes Dev.

[B60] Teleman AA, Chen YW, Cohen SM (2005). Drosophila Melted modulates FOXO and TOR activity. Dev Cell.

[B61] Jacinto E, Loewith R, Schmidt A, Lin S, Ruegg MA, Hall A, Hall MN (2004). Mammalian TOR complex 2 controls the actin cytoskeleton and is rapamycin insensitive. Nat Cell Biol.

[B62] Loewith R, Hall MN (2004). TOR Signaling in Yeast: Temporal and Spatial Control of Cell Growth. Cell Growth.

[B63] Hannan KM, Brandenburger Y, Jenkins A, Sharkey K, Cavanaugh A, Rothblum L, Moss T, Poortinga G, McArthur GA, Pearson RB (2003). mTOR-dependent regulation of ribosomal gene transcription requires S6K1 and is mediated by phosphorylation of the carboxy-terminal activation domain of the nucleolar transcription factor UBF. Mol Cell Biol.

[B64] Yuan X, Zhou Y, Casanova E, Chai M, Kiss E, Grone HJ, Schutz G, Grummt I (2005). Genetic inactivation of the transcription factor TIF-IA leads to nucleolar disruption, cell cycle arrest, and p53-mediated apoptosis. Mol Cell.

[B65] Jorgensen P, Rupes I, Sharom JR, Schneper L, Broach JR, Tyers M (2004). A dynamic transcriptional network communicates growth potential to ribosome synthesis and critical cell size. Genes Dev.

[B66] Browne GJ, Proud CG (2004). A novel mTOR-regulated phosphorylation site in elongation factor 2 kinase modulates the activity of the kinase and its binding to calmodulin. Mol Cell Biol.

[B67] Caldarola S, Amaldi F, Proud CG, Loreni F (2004). Translational regulation of terminal oligopyrimidine mRNAs induced by serum and amino acids involves distinct signaling events. J Biol Chem.

[B68] Chung J, Grammer TC, Lemon KP, Kazlauskas A, Blenis J (1994). PDGF- and insulin-dependent pp70S6k activation mediated by phosphatidylinositol-3-OH kinase. Nature.

[B69] Radimerski T, Montagne J, Hemmings-Mieszczak M, Thomas G (2002). Lethality of Drosophila lacking TSC tumor suppressor function rescued by reducing dS6K signaling. Genes Dev.

[B70] Weng QP, Andrabi K, Klippel A, Kozlowski MT, Williams LT, Avruch J (1995). Phosphatidylinositol 3-kinase signals activation of p70 S6 kinase in situ through site-specific p70 phosphorylation. Proc Natl Acad Sci USA.

[B71] Inoki K, Li Y, Zhu T, Wu J, Guan KL (2002). TSC2 is phosphorylated and inhibited by Akt and suppresses mTOR signalling. Nat Cell Biol.

[B72] Manning BD, Tee AR, Logsdon MN, Blenis J, Cantley LC (2002). Identification of the tuberous sclerosis complex-2 tumor suppressor gene product tuberin as a target of the phosphoinositide 3-kinase/akt pathway. Mol Cell.

[B73] Potter CJ, Pedraza LG, Xu T (2002). Akt regulates growth by directly phosphorylating Tsc2. [see comments.]. Nature Cell Biology.

[B74] Dong J, Pan D (2004). Tsc2 is not a critical target of Akt during normal Drosophila development. Genes Dev.

[B75] Bettencourt-Dias M, Giet R, Sinka R, Mazumdar A, Lock WG, Balloux F, Zafiropoulos PJ, Yamaguchi S, Winter S, Carthew RW (2004). Genome-wide survey of protein kinases required for cell cycle progression. Nature.

[B76] March JC, Bentley WE (2004). Insulin stimulates double-stranded RNA uptake in Drosophila S2 cells. Biotechniques.

[B77] Wang X, Beugnet A, Murakami M, Yamanaka S, Proud CG (2005). Distinct signaling events downstream of mTOR cooperate to mediate the effects of amino acids and insulin on initiation factor 4E-binding proteins. Mol Cell Biol.

[B78] Böhni R, Riesgo-Escovar J, Oldham S, Brogiolo W, Stocker H, Andruss BF, Beckingham K, Hafen E (1999). Autonomous control of cell and organ size by CHICO, a Drosophila homolog of vertebrate IRS1-4. Cell.

[B79] Scanga SE, Ruel L, Binari RC, Snow B, Stambolic V, Bouchard D, Peters M, Calvieri B, Mak TW, Woodgett JR (2000). The conserved PI3'K/PTEN/Akt signaling pathway regulates both cell size and survival in Drosophila. Oncogene.

[B80] Verdu J, Burtovich MA, Wilder EL, Birnbaum MJ (1999). Cell autonomous regulation of cell and organ growth in Drosophila by AKT/PKB. Nature Cell Biology.

[B81] Weinkove D, Neufeld TP, Twardzik T, Waterfield MD, Leevers SJ (1999). Regulation of imaginal disc cell size, cell number and organ size by *Drosophila *class I_A _phosphoinositide 3-kinase and its adaptor. Current Biology.

[B82] Hernandez G, Altmann M, Sierra JM, Urlaub H, del Corral RD, Schwartz P, Rivera-Pomar R (2005). Functional analysis of seven genes encoding eight translation initiation factor 4E (eIF4E) isoforms in Drosophila. Mech Dev.

[B83] Clemens JC, Worby CA, Simonson-Leff N, Muda M, Maehama T, Hemmings BA, Dixon JE (2000). Use of double-stranded RNA interference in Drosophila cell lines to dissect signal transduction pathways. Proc Natl Acad Sci USA.

[B84] Grewal SS, Li L, Orian A, Eisenman RN, Edgar BA (2005). Myc-dependent regulation of ribosomal RNA synthesis during Drosophila development. Nat Cell Biol.

