# Supplementary figures and images for: Rheb-TOR signaling promotes protein synthesis, but not glucose or amino acid import, in Drosophila
Source: BMC Biol. 2007 Mar 19;5:10. doi: 10.1186/1741-7007-5-10 (PMC1847425; doi:10.1186/1741-7007-5-10)

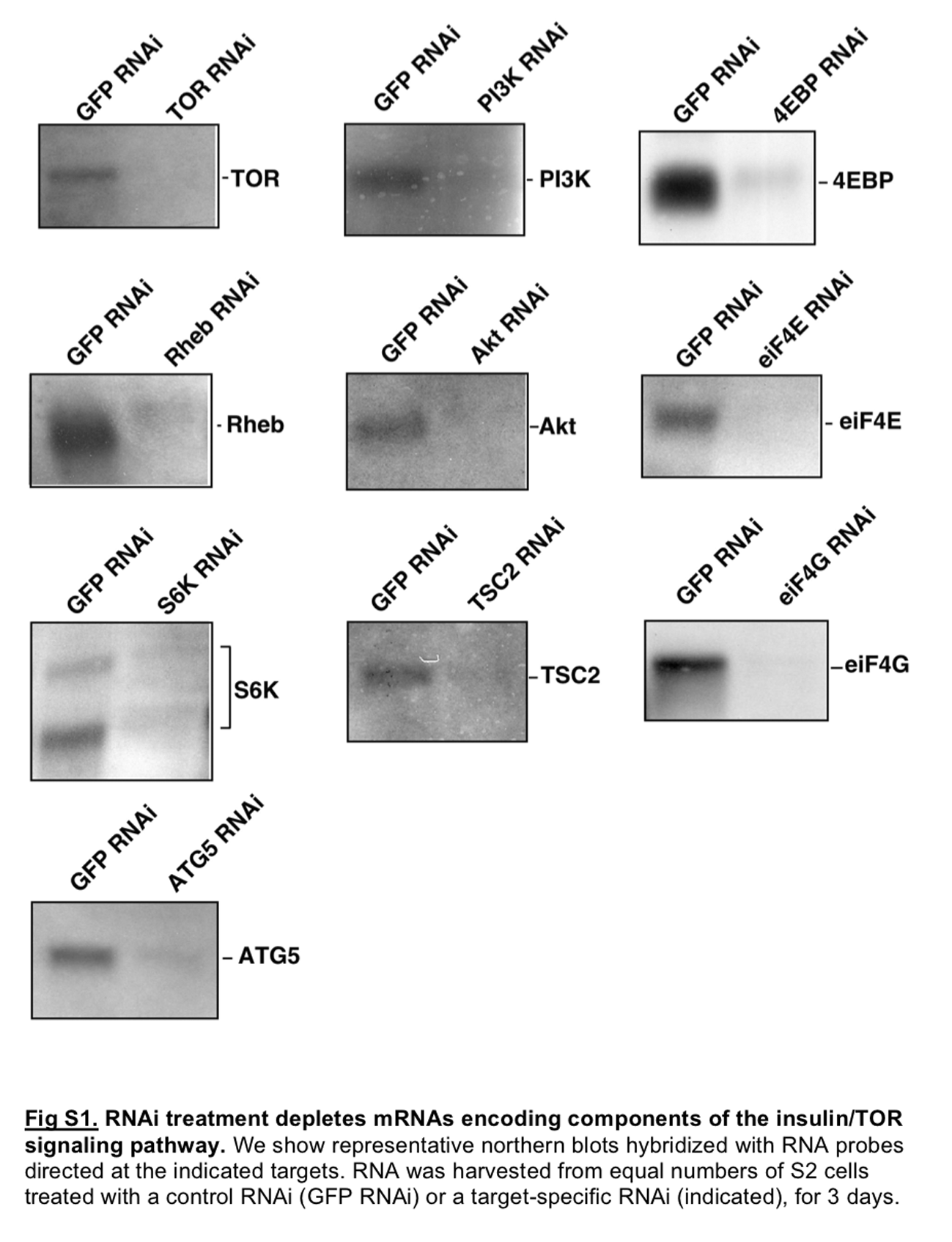

Supplement: Additional file 1 — Supplementary Figure 1: RNAi treatment depletes mRNAs encoding components of the insulin/TOR signaling pathway. We show representative northern blots hybridized with RNA probes directed at the indicated targets. RNA was harvested from equal numbers of S2 cells treated with a control RNAi (GFP RNAi) or a target specific RNAi (indicated), for three days. [file 1741-7007-5-10-S1.tiff]
